# Editorial: Innovative strategies for improving sperm lifespan during storage

**DOI:** 10.3389/fvets.2026.1920660

**Published:** 2026-07-27

**Authors:** Francisco Alberto García-Vázquez, Jose Luis Ros-Santaella, Federica Turri, Eliana Pintus

**Affiliations:** 1 Department of Physiology, Faculty of Veterinary Science, University of Murcia, Murcia, Spain; 2 Department of Veterinary Sciences, Faculty of Agrobiology, Food and Natural Resources, Czech University of Life Sciences Prague, Prague, Czechia; 3 Institute of Agricultural Biology and Biotechnology (IBBA), Italian National Research Council (CNR), Lodi, Italy

**Keywords:** antioxidants, ferroptosis, imaging flow cytometry, metabolomics, oxidative stress, semen extenders, semen storage, sperm cryopreservation

Sperm storage is a fundamental process in mammalian reproduction, occurring both *in vivo* and *in vitro*. *In vivo*, spermatozoa acquire fertilizing competence and remain stored for variable periods within the epididymis before ejaculation (1), and, after mating or artificial insemination, they may also persist transiently in the female reproductive tract (2). *In vitro*, this biological principle is applied through semen preservation technologies, mainly refrigerated storage for short-term use and cryopreservation for long-term preservation. These approaches are now routine components of artificial insemination programs, genetic resource banking, and conservation strategies (3–5). Nevertheless, spermatozoa are highly specialized cells with limited capacity for repair, and their survival outside the reproductive tract remains technically challenging.

During semen handling, refrigerated storage, and cryopreservation, sperm cells are exposed to several external stressors, including dilution, cold and heat shock, osmotic imbalance, oxidative damage, mechanical injury, and chemical toxicity (6). Among them, oxidative stress is one of the major mechanisms underlying sperm deterioration during storage. Although low levels of reactive oxygen species (ROS) are required for normal sperm function, excessive ROS production during semen handling or storage can overwhelm the limited antioxidant defenses of spermatozoa (7). This imbalance promotes lipid peroxidation, mitochondrial dysfunction, DNA damage, and activation of regulated cell death pathways, ultimately compromising motility and fertilizing capacity (8). Accordingly, antioxidant supplementation and strategies aimed at preserving redox homeostasis have become central approaches for improving sperm survival during storage.

This Research Topic brings together studies describing new cryoprotective strategies, metabolomic insights, and advanced sperm assessment technologies aimed at improving the efficiency of semen preservation, particularly in livestock species of reproductive interest, including small and large ruminants and pigs.

A major theme emerging from this Research Topic is that sperm damage during storage cannot be explained only by physical injury. Rather, it also involves molecular responses that link redox imbalance with mitochondrial impairment, membrane destabilization, and regulated cell death pathways. Related to the last, apoptosis and ferroptosis (an iron-dependent, lipid peroxidation-driven form of regulated cell death) are good indicators of sperm cryopreservation injury (9). It has been shown that cryopreservation increases the apoptotic marker cleaved-caspase-3 and maintains high expression of the ferroptosis-related marker transferrin receptor (Hai et al.). Moreover, the use of ferroptosis inhibitors improves post-thaw sperm quality, with ferrostatin-1 showing the strongest protective effect by reducing ROS, lipid peroxidation, and Fe2 + levels, while restoring glutathione peroxidase 4 (GPX4) expression (a key regulator of ferroptosis) to values comparable with those of fresh sperm (Hai et al.). This study is especially relevant because it places ferroptosis within the mechanistic framework of sperm cryoinjury.

Among the different approaches presented in this Research Topic, several studies explored antioxidant-based strategies to improve sperm cryosurvival. In this sense, docosahexaenoic acid (DHA) has been shown to significantly influence the structure and function of mammalian cells (10), including spermatozoa (11, 12). The supplementation of yak semen extenders with DHA before cryopreservation improved post-thaw sperm quality in a concentration-dependent manner, with 10 ng/mL identified as the optimal dose (Zhu et al.). At this concentration DHA enhanced total and progressive motility, kinematic parameters, membrane and acrosome integrity, mitochondrial membrane potential, and ATP levels (Zhu et al.). These benefits were accompanied by lower ROS and malondialdehyde (MDA) concentrations, enhanced antioxidant enzyme activities, and reduced activation of apoptosis-related pathways (Zhu et al.).

The search for new cryoprotective strategies is particularly relevant in boar semen, since the use of frozen-thawed sperm for swine artificial insemination remains limited by its lower efficiency compared with liquid-stored semen (13, 14). Boar spermatozoa are highly sensitive to cooling and freezing damage, partly because of their membrane lipid composition and susceptibility to oxidative stress (15, 16). In this context, natural compounds have emerged as promising additives for semen extenders, as they may contribute to antioxidant and antimicrobial activities while helping to preserve sperm function during storage (17). Mangiferin is a natural C-glycosylxanthone polyphenol found in traditional Chinese medicinal plants and exhibits various biological effects, including antioxidant activity (18). The cryoprotective potential of mangiferin was evaluated in boar semen by adding this compound to freezing extenders (Zhang et al.). A concentration of 10 μM showed the strongest protective effect, improving post-thaw sperm quality. These improvements were associated with increased glutathione (GSH) levels and total antioxidant capacity, together with reduced ROS, and MDA concentrations. Complementing this approach, other authors have evaluated whether combining two antioxidants hesperidin, a citrus-derived flavonoid, and ascorbic acid, or vitamin C, could provide additional protection during boar semen cryopreservation (Dong et al.). The optimal combination, 15 μM hesperidin plus 100 μM ascorbic acid, provided greater protection than either compound alone, improving overall post-thaw sperm quality while reducing oxidative stress and apoptosis.

Beyond the development of protective additives, improving semen preservation also requires a better understanding of the biological factors of sperm survival during storage. In this regard, omics approaches (19) (Bodu et al.; Shi et al.) are being used to identify molecular biomarkers. Specifically, the identification of metabolites involved in natural sperm maturation and storage may provide useful clues for improving *in vitro* preservation systems. This is particularly relevant for the epididymis, where spermatozoa acquire fertilizing competence and remain stored until ejaculation (20). Accordingly, the epididymal environment can serve as a biological reference for designing more physiological extenders adapted to the metabolic requirements of spermatozoa. Untargeted LC-MS metabolomics was used to characterize metabolic differences among the caput, corpus, and cauda epididymis of Hu sheep, revealing region-specific metabolic profiles associated with sperm maturation and epididymal storage (Shi et al.). Although this study did not directly evaluate cryopreservation, metabolites such as choline and L-carnitine were identified as potential targets for improving semen preservation strategies. In support of this idea, the addition of L-carnitine to cryopreservation media has been shown to be beneficial for spermatozoa in different species (21–23). Thus, the identification of metabolites associated with sperm storage also fits within the broader use of omics technologies in semen preservation that can reveal molecular biomarkers related to sperm quality and freezability (Bodu et al.). These approaches are particularly valuable because sperm tolerance to storage varies among species, breeds, individuals, and even ejaculates (Bodu et al.). Therefore, integrating omics information may help explain these differences and guide the design of more targeted extenders, adapted to the specific biological requirements of each species or genetic background.

While omics approaches can help identify biological targets for improving semen preservation, their practical translation also requires tools capable of optimizing and evaluating preservation protocols more efficiently. In this sense, artificial intelligence is beginning to be used as a complementary tool to analyze complex datasets and support decision-making in reproduction in general, and semen cryopreservation in particular (24). For example, machine learning has been applied to optimize bull semen extender formulations (25). Similar computational approaches may also improve the evaluation of sperm quality by reducing subjectivity and increasing analytical throughput. This is especially relevant when combined with advanced image-based platforms, such as imaging flow cytometry, which integrates the quantitative power of flow cytometry with morphological information from microscopy (Umirbaeva et al.). In this regard, label-free imaging flow cytometry combined with deep learning was applied to bovine sperm morphology analysis in fresh and cryopreserved samples, allowing automated and high-throughput classification of different morphological categories, with the best performing model reaching an accuracy of 91.1% (Umirbaeva et al.).

Taken together, this Research Topic provides complementary studies addressing strategies to improve sperm survival during storage, from the modulation of oxidative stress and regulated cell death pathways to metabolomic characterization, extender design, and automated sperm assessment. The integration of these complementary approaches will contribute to the development of more efficient and species-specific preservation strategies.
